# Supplementary material for: Coordinated Representational Drift Across the Mouse Cortex
Source: Res Sq. 2026 Jun 11:rs.3.rs-9933652. Preprint. [Version 1] doi: 10.21203/rs.3.rs-9933652/v1 (PMC13278330; doi:10.21203/rs.3.rs-9933652/v1)
Supplement: Supplement 1 [file NIHPPrs9933652v1-supplement-1.pdf]

B Supplementary

B.1 Spatial consistency increases with time

One repeated pattern we observed in the single-cell LSTFs was that cell tuning would tighten and/or become more consistent over time (**Supplementary Fig. 3**). To quantify this change in spatial representations across the 47-day recording period, we correlated individual lap tuning functions with each neuron's mean tuning across all sessions, and observed the change in correlation across time. The lap-wise correlation increased from early sessions (session 1: 0.324 $\pm$ 0.059 SD across mice) to late sessions (session 14: 0.435 $\pm$ 0.074) (**Supplementary Fig. 3B**).

This increase in consistency was observed across all cortical regions, and in each individual mouse. From session 1 to session 14, across all mice, visual cortex consistency increased from 0.369 $\pm$ 0.059 SD to 0.458 $\pm$ 0.064, retrosplenial cortex from 0.324 $\pm$ 0.085 to 0.452 $\pm$ 0.090, somatosensory cortex from 0.320 $\pm$ 0.058 to 0.440 $\pm$ 0.065, and motor cortex from 0.315 $\pm$ 0.066 to 0.414 $\pm$ 0.047. The consistent increase across all examined regions indicated that spatial tuning became more reliable with continued experience in this behavioral paradigm.

B.2 Linear decoding of mouse position

To assess whether mouse position could be linearly decoded both within and across recording sessions, we trained linear-kernel support vector machine classifiers (`sklearn.svm.SVC` with default $C = 1.0$ and one-vs-one multi-class strategy) to predict spatial bin identity from population activity. Each training sample consisted of the population activity vector at one spatial bin on one lap, drawn from the lapwise spatial tuning functions, with the corresponding bin index $(1, \dots, 36)$ as the target label. For intra-session decoding, we used leave-one-lap-out cross-validation within each session. For inter-session decoding, we trained on all laps of one session and evaluated on each lap of another session, restricting both sessions to neurons that were successfully tracked across the pair. At test time, predicted bin indices were converted to two-dimensional spatial coordinates by mapping each bin to its centroid (the mean mouse position within that bin), and performance was quantified as the mean of the R^2 scores for the x and y coordinates.

Within-session decoding performance was highly reliable across all mice (R^2 = 0.921 $\pm$ 0.040 SD across mice), with individual mice showing consistent spatial decoding accuracy (R^2 range: 0.838 to 0.976). Brain region analysis revealed that visual (VIS) and retrosplenial (RSP) areas provided the strongest decoding performance both individually (VIS: 0.895 $\pm$ 0.042 R^2; RSP: 0.889 $\pm$ 0.066 R^2) and in combination (VIS + RSP: 0.917 $\pm$ 0.049 R^2), while somatosensory regions showed more variable performance (SSp: 0.706 $\pm$ 0.105 R^2; MO: 0.639 $\pm$ 0.285 R^2), which aligns with our spatial consistency results.

Cross-session decoding revealed characteristic temporal dynamics in representation stability. When decoding between consecutive sessions (Δt = 1), performance remained high across most session pairs (R^2 = 0.864 $\pm$ 0.081 SD across mice). However, decoding performance gradually declined as the temporal separation between training and test sessions increased, with sessions separated by maximum intervals showing substantially reduced accuracy (**Supplementary Fig. 2**, R^2 = 0.223 $\pm$ 0.185 when Δt = 13). Interestingly, individual mice exhibited distinct patterns, with decoding performance clustering into two groups (**Supplementary Fig. 2B**) that maintained consistent relative performance across session pairs.

B.3 Preserved covariation of population embeddings under drift implies orthogonality

The finding that position-by-position cross-correlation matrices are preserved across sessions raises the question of whether the inter-session transformation must itself be orthogonal. We show that this is true on the representational subspace, provided population norms scale uniformly across sessions. This provides theoretical justification for the Procrustes analysis and explains why rotation-like dynamics are consistently observed in representational drift studies.

Let $\mathbf{x}_s^i \in \mathbb{R}^n$ denote the population activity vector for position bin $i \in \{1, \ldots, m\}$ in session s, with $m = 36$ and n the number of neurons. Let $X_s = [\mathbf{x}_s^1, \ldots, \mathbf{x}_s^m] \in \mathbb{R}^{n\times m}$ denote the matrix of population vectors, and let $\mathcal{V} = \mathrm{span}(\{\mathbf{x}_s^1, \ldots, \mathbf{x}_s^m\}) \subseteq \mathbb{R}^n$ denote the representational subspace.

We make the following assumptions:

1. *Correlation preservation:* $\mathrm{corr}(\mathbf{x}_s^i, \mathbf{x}_s^j) = \mathrm{corr}(\mathbf{x}_t^i, \mathbf{x}_t^j)$ for all position bins i, j.
2. *Linear inter-session transformation:* there exists $R \in \mathbb{R}^{n\times n}$ such that $\mathbf{x}_t^i = R\mathbf{x}_s^i$ for all i.
3. *Uniform norm scaling:* $\|\mathbf{x}_t^i\| = c\|\mathbf{x}_s^i\|$ for all i, where $c > 0$ is constant.

Under these assumptions, the restriction of R to the representational subspace satisfies

$$R^\top R = c^2 I \quad \text{on } \mathcal{V}$$

so R acts as an orthogonal transformation on $\mathcal{V}$ up to the scale factor c (and is exactly orthogonal on $\mathcal{V}$ when $c = 1$).

Proof

First, we express the correlation preservation condition where for any position bins i and j:

$$\text{corr}(\mathbf{x}_s^i, \mathbf{x}_s^j) = \frac{\mathbf{x}_s^{i\top}\mathbf{x}_s^j}{\|\mathbf{x}_s^i\|\|\mathbf{x}_s^j\|}$$

$$\text{corr}(\mathbf{x}_t^i, \mathbf{x}_t^j) = \frac{\mathbf{x}_t^{i\top}\mathbf{x}_t^j}{\|\mathbf{x}_t^i\|\|\mathbf{x}_t^j\|} = \frac{(R\mathbf{x}_s^i)^\top(R\mathbf{x}_s^j)}{\|R\mathbf{x}_s^i\|\|R\mathbf{x}_s^j\|} = \frac{\mathbf{x}_s^{i\top}R^\top R\mathbf{x}_s^j}{\|R\mathbf{x}_s^i\|\|R\mathbf{x}_s^j\|}$$

Then, by our first assumption

$$\frac{\mathbf{x}_s^{i\top}R^\top R\mathbf{x}_s^j}{\|R\mathbf{x}_s^i\|\|R\mathbf{x}_s^j\|} = \frac{\mathbf{x}_s^{i\top}\mathbf{x}_s^j}{\|\mathbf{x}_s^i\|\|\mathbf{x}_s^j\|}$$

Next, we use the uniform norm scaling assumption that says that $\|R\mathbf{x}_s^i\| = c\|\mathbf{x}_s^i\|$ for all i. Therefore

$$\frac{\|R\mathbf{x}_s^i\|\|R\mathbf{x}_s^j\|}{\|\mathbf{x}_s^i\|\|\mathbf{x}_s^j\|} = \frac{c\|\mathbf{x}_s^i\| \cdot c\|\mathbf{x}_s^j\|}{\|\mathbf{x}_s^i\|\|\mathbf{x}_s^j\|} = c^2$$

Substituting into the equation from the first step we get

$$\frac{\mathbf{x}_s^{i\top} R^\top R \mathbf{x}_s^j}{c^2 \|\mathbf{x}_s^i\| \|\mathbf{x}_s^j\|} = \frac{\mathbf{x}_s^{i\top} \mathbf{x}_s^j}{\|\mathbf{x}_s^i\| \|\mathbf{x}_s^j\|}$$

Therefore

$$\mathbf{x}_s^{i\top} R^\top R \mathbf{x}_s^j = c^2 \mathbf{x}_s^{i\top} \mathbf{x}_s^j \quad \forall i, j$$

Which can be rewritten in matrix form as

$$X_s^\top R^\top R X_s = c^2 X_s^\top X_s$$

Finally, we need to show that $R^\top R = c^2 I$ on the representational subspace.
To do so, note that for any vector $\mathbf{v} \in \mathcal{V}$, we can write $\mathbf{v} = X_s \boldsymbol{\alpha}$ for some $\boldsymbol{\alpha} \in \mathbb{R}^m$. Then

$$\mathbf{v}^\top R^\top R \mathbf{v} = \boldsymbol{\alpha}^\top X_s^\top R^\top R X_s \boldsymbol{\alpha} = c^2 \boldsymbol{\alpha}^\top X_s^\top X_s \boldsymbol{\alpha} = c^2 \mathbf{v}^\top \mathbf{v}$$

This shows that $\|R\mathbf{v}\|^2 = c^2 \|\mathbf{v}\|^2$ for all $\mathbf{v} \in \mathcal{V}$. By the polarization identity we have

$$\mathbf{u}^\top R^\top R \mathbf{v} = \frac{1}{4} \left(\|R(\mathbf{u}+\mathbf{v})\|^2 - \|R(\mathbf{u}-\mathbf{v})\|^2 \right) = \frac{c^2}{4} \left(\|\mathbf{u}+\mathbf{v}\|^2 - \|\mathbf{u}-\mathbf{v}\|^2 \right) = c^2 \mathbf{u}^\top \mathbf{v}$$

for all $\mathbf{u}, \mathbf{v} \in \mathcal{V}$.
Therefore $R^\top R = c^2 I$ on $\mathcal{V}$. $\square$

C Supplementary Figures

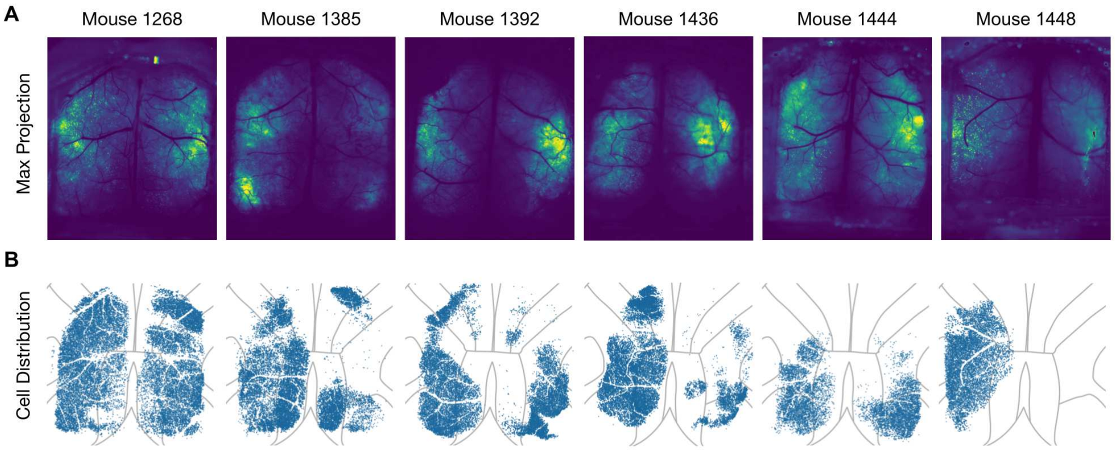

Supplementary Figure 1: Imaging fields of view and anatomical distribution of tracked neurons across mice. (A) Maximum-intensity projections from session 1 of each mouse, produced by Suite2p. **(B)** Anatomical distribution of tracked neurons in each mouse, overlaid on the Allen Mouse Brain Atlas.

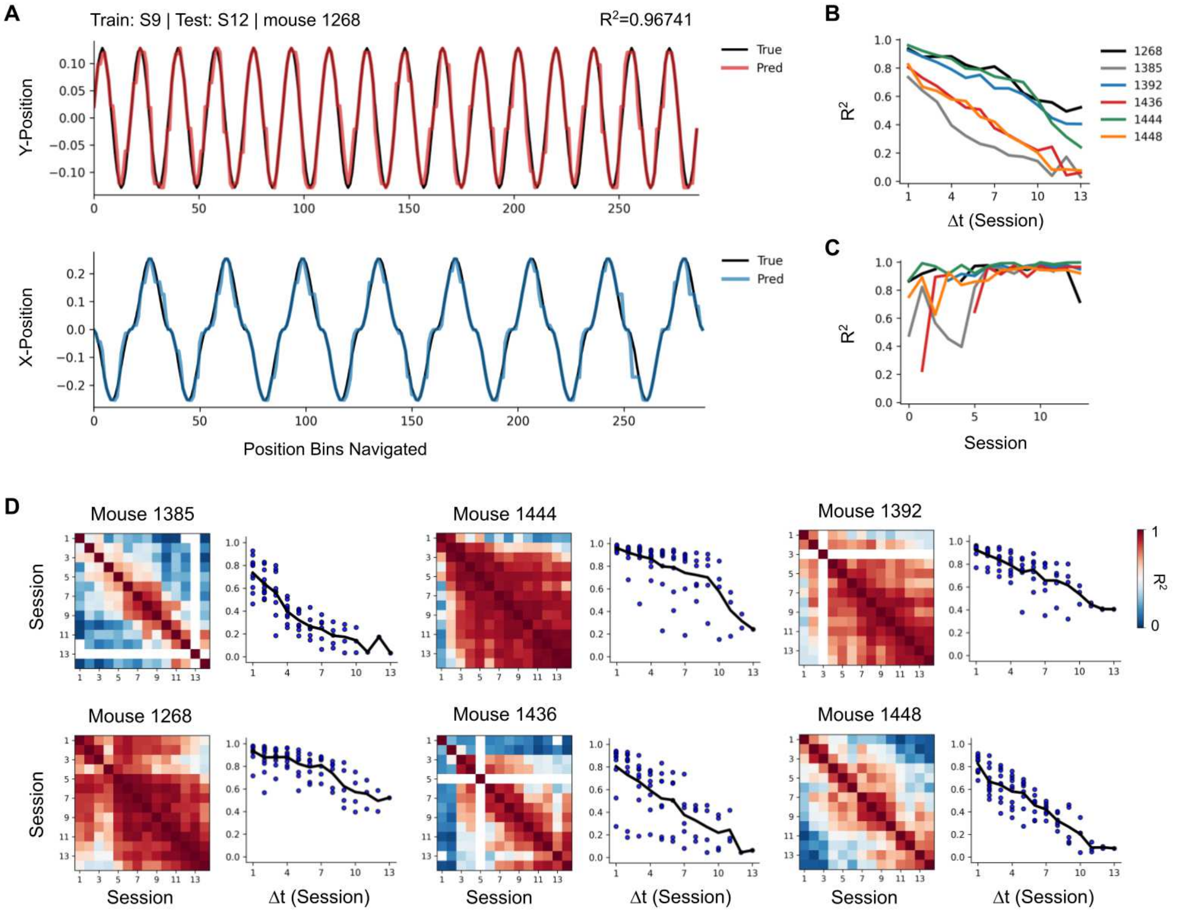

Supplementary Figure 2: Linear decoding of mouse position from population activity within and across sessions. **(A)** Example cross-session decoding performance for X (blue) and Y (red) axes using mouse 1268, with a decoder trained on session 9 and evaluated on session 12. **(B)** Mean inter-session decoding performance versus session separation shown for each mouse. **(C)** Mean intra-session decoding performance within each session shown for each mouse. **(D)** Cross-session decoding performance matrices for each mouse. Dark red indicates high performance (R^2=1), while dark blue indicates low performance (R^2=0). Adjacent to each matrix is the decoding performance as a function of session separation.

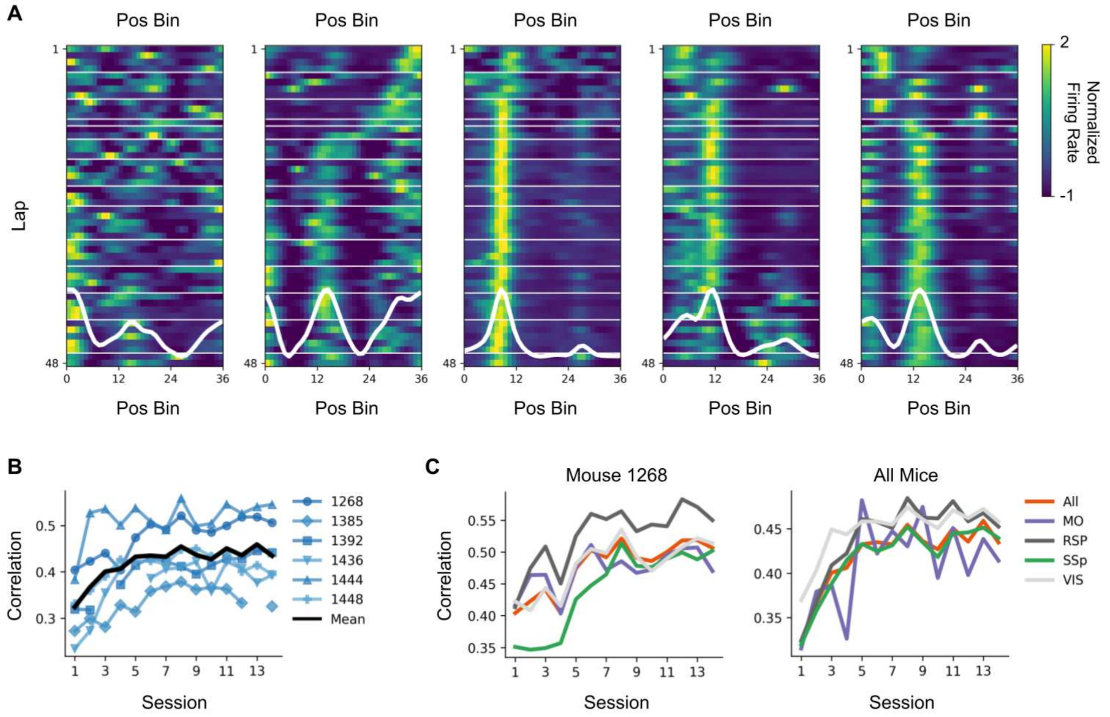

Supplementary Figure 3: Spatial tuning consistency increases over the recording period across cortical regions. **(A)** Sample LSTFs from mouse 1268 that show this increase in consistency over time. **(B)** Correlation to the mean tuning averaged across all neurons for each mouse, then averaged across all mice shown in black. **(C)** The correlation to the mean tuning averaged across all neurons in each brain region for mouse 1268 (left) and all mice (right).
